# Exploring the working life of people with multiple sclerosis during the COVID-19 pandemic in Sweden

**DOI:** 10.1186/s12889-024-18844-9

**Published:** 2024-05-23

**Authors:** Chantelle Murley, Jessica Dervish, Alejandra Machado, Veronica Svärd, Agneta Wennman-Larsen, Jan Hillert, Emilie Friberg

**Affiliations:** 1 Division of Insurance Medicine, Department of Clinical Neuroscience, Karolinska Institutet, Stockholm, SE-171 77 Sweden; 2 Department of Social Work, School of Social Sciences, Huddinge, SE-141 89 Sweden; 3 Department of Nursing Science, Sophiahemmet University, Stockholm, SE-114 86 Sweden; 4 Division of Neurology, Department of Clinical Neuroscience, Karolinska Institutet, Stockholm, SE-171 77 Sweden

**Keywords:** SARS-CoV-2, Containment measures, Work, Employment, Occupation, Chronic disease, Remote work

## Abstract

**Background:**

The COVID-19 pandemic led to vast changes in working life and conditions in which we work. These changes may affect people with multiple sclerosis (PwMS) differently. We aimed to describe the working situation of PwMS during the COVID-19 pandemic and the pandemic’s impact on their working lives.

**Methods:**

All individuals aged 20–50 listed in the Swedish Multiple Sclerosis Registry were invited to participate in an online survey in 2021. Closed and open-ended responses linked to individual-level register data were used in this exploratory mixed-methods study. Differences in the proportions reporting specific impacts were assessed with chi-square tests by sex, MS severity, education, and profession. The open-ended answers were analysed through content analysis.

**Results:**

Over 8500 PwMS were invited (52% response rate). We included the 3887 respondents who answered questions about the impact of the pandemic on working life. Most (93.7%) reported being in paid work. An impact of the ongoing pandemic to one’s daily occupation was reported by 26.2%, with different characteristics observed across the impacts. Four categories of type of answers were identified from the open-ended answers: Direct impact on one’s occupation, Disclosing or concealing MS in the workplace, Worry and uncertainty, and Broader impact to life situation.

**Conclusions:**

PwMS navigated the pandemic by interrupting as well as continuing their working lives. Many PwMS reported that the pandemic did not affect their work situation. However, the reported impacts differed among the participants and a sense of uncertainty and worry was often underlying their statements. Lessons from the pandemic may support future work participation.

## Introduction

Public health interventions were rapidly implemented to reduce virus transmission during the COVID-19 pandemic. The impact of the pandemic and the consequent interventions on one’s working life is of interest given that work is an important dimension of life [1]. The consequences for work participation and conditions of work may differ among people with chronic disease, such as people with multiple sclerosis (PwMS).

An additional source of uncertainty PwMS faced during the pandemic related to their multiple sclerosis (MS), their treatments, and pre-existing activity limitations [2]. The Public Health Agency of Sweden recommended that persons with neurological disorders should be especially cautious and physically distance but provided no MS-specific recommendations [3]. The Swedish MS Society suggested additional precautions following treatment administration based on possible treatment-specific risk profiles [4]. Older age, comorbidities, disability, and progressive MS have emerged to be associated with more severe COVID-19 outcomes among PwMS [3, 5–7].

Accordingly, the pandemic may exacerbate the wider challenges PwMS often face, such as maintaining work [2]. These challenges are in part owing to the varying symptoms of MS and uncertain disease course. Pre-pandemic knowledge shows that changes in working life occur early for PwMS [8–10], and have found that a lower proportion of PwMS are employed [8]. Sweden has a higher proportion of PwMS in paid work than other European countries [11]. However, they often work part-time alongside part-time social insurances [9, 12]. Therefore, it is of interest to investigate how people with this chronic and often invisible disease have coped during the pandemic.

Response strategies to the new virus ranged from suppression to mitigation, with differing levels of population and venue specificity [13]. Many countries introduced periods of lockdown and enforced mandates [14]. Whereas Sweden largely applied their pre-existing pandemic plan to reduce virus transmission over a longer time horizon and prevent the healthcare system from collapsing [13, 15, 16]. With this mitigation strategy, restrictions on certain forms of activity (e.g., capacity limits) were combined with public health recommendations [13, 16, 17]. Recommendations included taking personal responsibility to physically distance, limit gatherings and social contacts, and to self-isolate when symptomatic [15, 18]. Several allowances and social insurances nudged behaviour in line with the recommendations, including a compensation to financially assist individuals isolating after virus exposure and the usual sickness absence benefits. A new earnings compensation was established for individuals in defined risk groups and their household members to isolate and prevent exposure. The Swedish National Board of Health and Welfare defined the risk groups, originally including MS [4, 19]. However, these definitions were soon revised to only include PwMS with severe motor disabilities or impaired respiratory function [20].

The general public was recommended to work from home, if possible, and many rapidly changed to distance work. However, Sweden had relatively many working onsite as businesses often stayed open with adaptations and distancing in place. Anecdotal reports suggest that PwMS raised questions about work and self-isolation with their neurologists; as MS was not in itself eligible for the compensation, the advice was often to reach agreement with one’s employer to work remotely [19]. However, individuals have different opportunities for requesting such changes and feasibility of working remotely. Furthermore, not all are willing to disclose their MS in the workplace [21]. The pandemic provided unique challenges; for managers to maintain safe workplaces [14] as well as for workers. We focus in the present study on how the pandemic affected workers with a chronic disease, namely MS. As lockdowns to suppress infection rates are costly, tiring, and cannot be sustained long-term, knowledge about less restrictive interventions is needed to collaboratively learn from the different responses [17].

Accordingly, our aim was to describe the working situation of PwMS during the COVID-19 pandemic and the self-reported impact of the pandemic on their working lives.

## Materials and methods

An exploratory mixed-method study was conducted based on a cross-sectional survey of PwMS in Sweden. Data collection was conducted from May to September 2021 and Statistics Sweden provided the dataset in October 2021. Substantial data management preceded the commencement of data analysis in the spring of 2022.

### Study population

All individuals living in Sweden who were aged 20–50 years and included in the Swedish Multiple Sclerosis Registry (SMSreg) were invited to participate in an online survey administered by Statistics Sweden. Of those invited, 4412 (52%) answered the survey, which had four reminders. The present study includes the 3887 participants without full-time disability pension and who answered either question directly related to the impact of the pandemic on working life.

### Data sources

This study integrated both quantitative and qualitative data. Of the 66 questions in the survey, this study utilises responses from close and open-ended questions on working life and the impact of the pandemic on their occupation. The questionnaire data was linked on an individual-level register data [22], to clinical data from the SMSreg [23], and sociodemographic data from the Longitudinal Integrated Database for Health Insurance and Labour Market Studies (LISA) [24]. Statistics Sweden performed the linkage and delivered anonymised data to the researchers.

### Outcomes

Responses to two specific close-ended questions on the impact of the pandemic on working life informed the study outcomes.

First, the impact of the COVID-19 pandemic was investigated among all participants by the question “*Has your employment been affected by the ongoing COVID-19 pandemic?*” with multiple responses possible: No; Yes, I am furloughed; Yes, I have been furloughed; Yes, I am unemployed (with compensation); Yes, I am unemployed (without compensation); Yes, I am employed and have had less to do; Yes, I am employed and have had more to do; Yes, I have started to study or pursue further education; Yes, other.

The two furlough and unemployed responses were merged respectively due to small numbers.

Second, self-employed PwMS were asked “*How has the ongoing COVID-19 pandemic affected you in respect of being self-employed?*”, with the possibility of responding: yes, partly, or no to the following statements: My business has gone better than before; I have mostly been able to work as usual; I have digitalised my business activities; I have undertaken other types of assignment; My business has gone down, but I have managed to keep it going; The business is dormant; I have dissolved my company; My business has gone into bankruptcy.

The yes and partly responses were collapsed.

Lastly, the survey also explored a closed-ended question regarding change of employment owing to the pandemic (“*Has the COVID-19 pandemic permanently affected your occupation in any of the following ways? -* e.g. Change of job”). However, due to the low percentages of PwMS confirming a change of employment, only qualitative aspects are presented.

In addition to the closed-ended questions, all participants could also describe other impacts in an open-ended response to the question asking about the impact of the pandemic on daily occupation. Moreover, the participants themselves raised the impact of the pandemic on their working life in other questions in the survey. Four questions with such responses concerned disclosure (*“Please share the main reason for why you have chosen to tell”*) or concealment (“*Please share the main reason for why you have chosen not to tell*”) of MS at work, as well as the consequences of this (“*What have been the positive or negative consequences of telling about MS at the workplace*?” and a fifth question “*What have been the positive or negative consequences of not telling about MS at the workplace*?” – which derived into two responses: positive and negative consequences registered separately). Relevant responses from the last survey question (“*Is there something more you would like to share in relation to your life and work situation that has not been addressed in the survey*?”) were also included. Accordingly, all responses from the six open-ended questions were used for the qualitative analyses if they related to the pandemic, COVID-19, or distance work.

### Covariates

Covariates regarding the participant’s working life were informed from the following survey questions.

Current occupation was assessed by asking: “*What is your current occupation*?”. Participants could report multiple occupations from the following: Employed; Own company/business; Student/work experience placement; On parental leave; On sick leave; On disability pension (part-time); On a leave of absence; Job seeker/unemployed; Other.

In paid work (yes, no) was informed from the question “*What is your profession?*” where participants could either state their current profession in an open-ended response, or state that they were not in paid work. The responses were then categorised to inform the type of current profession considering the formal responsibility for personnel and level of physical demands (managerial responsibilities, low-medium physical demands, and high physical demands) as described elsewhere [21]. Importance of paid work (“*How important is paid work in your life?”*) was ranked on a five-point scale from one of the least important things to one of the most important.

The sociodemographic covariates from LISA were: sex; type of living area; level of education; family composition; and country of birth. Sociodemographic data was missing for seven participants not registered as living in Sweden on 31 December 2019.

Type of MS (relapsing-remitting, secondary-progressive, primary-progressive, missing) and MS severity, assessed with the most recent Expanded Disability Status Scale score (EDSS), were informed by the SMSreg. Scores were set to missing if the assessment was more than three years old (*n* = 441) or if the score was recorded as 0 but the participant had progressive MS (*n* = 15) [21]. EDSS scores were then categorised (mild disability, yes (0-2.5) or no (3-9.5)).

Lastly, years since MS diagnosis and age were calculated from the date of survey response and subtracting the date of diagnosis and date of birth, respectively.

### Statistical and qualitative data analyses

The study population was described with frequencies and proportions for the categorical covariates and median values with an interquartile range (IQR) for continuous covariates. Differences by sex were assessed with chi-square tests. In addition, descriptive statistics were calculated for the participants reporting self-employment.

The outcome measures were then tabulated by sex. Differences in the impacts to one’s occupation by sex, education level, type of current profession, MS severity, and self-employment were assessed with chi-square tests.

All statistical analyses were performed using SAS v.9.4 and a p-value of < 0.05 was considered statistically significant.

The participants’ responses to the open-ended questions served as the basis for the explorative qualitative analysis, using content analysis [25, 26]. The analysis aimed to complement and deepen the understanding of the quantitative analyses of the study questions by providing more detailed, contextualised, and complex views on how the pandemic impacted the working life of PwMS [27].

The qualitative study material included 204 open-ended answers to the survey question directly asking about the pandemic impact on one’s occupation, statements from the four questions regarding disclosure or concealment of their MS diagnosis in the workplace, and 68 statements relating to the pandemic from the last question asking about anything further to add. Regarding the disclosure or concealment statements, the first 1000 participants had statements from any of the four questions coded in detail. The remaining 2810 participants were coded only if the responses included new codes or added further rich descriptions to already existing codes. This material contributed to the analyses with reflections on the impact the pandemic has had on their working life.

NVivo v.11 supported the coding of the participants’ statements. First, one of the researchers (JD) carefully examined and iteratively coded the answers to the six open-ended questions separately. The coded data was organised first question-by-question and then at a later step after discussion with all researchers, the statements from all open-ended responses were analysed together as a single material. Through combination, the reoccurring content in the data could be condensed to arrive at the categories and sub-categories. Interpretation of the meaning of the included data was initially conducted (CM, JD, and EF) by organizing the codes and categories question-by-question to facilitate the analysis. Subsequent discussions (AM, VS, AWL, and JH) were conducted to verify the initial framework and draw meaningful conclusions across the gathered material to deepen understanding of the quantitative analysis.

## Results

### Study participants’ characteristics

The median age of the included 3887 participants was 41 years (IQR: 35–46) (Table 1). They had a median of 8.1 years since their MS diagnosis (IQR: 4.1–13.3, *n* = 3631) and nearly all (94.0%) had relapsing-remitting MS (data not shown). MS severity was predominantly mild (EDSS = 0-2.5; 68.0%), with 23.9% of the total sample having an EDSS score of 0. Moderate to severe MS (EDSS = 3–9,5) accounted for 11.9% of the sample, among whom only 2.0% had a score of 6 or higher. The remaining participants had missing EDSS information or the assessment was deemed too old (20.1%).

**Table 1:** Descriptive characteristics of all the study participants, as well as stratified by sex

		Sex^1^		*p*-value^2^	All study participants (*n* = 3887)
		Women (*n* = 2755)	Men (*n* = 1132)		
		*n*(%)	*n*(%)		*n*(%)
***Sociodemographic characteristics***					
Age (years)	20–29	288(10.5)	100(8.8)	0.488	388 (10)
	30–39	913(33.1)	378(33.4)		1291(33.2)
	40–49	1397(50.7)	585(51.8)		1983 (51)
	50+	157(5.7)	68(6.0)		225(5.8)
Age, median (IQR)		41 (35-46)	41 (35-46)		41 (35-46)
Type of living area	Cities	1221(44.4)	535(47.3)	0.189	1757(45.2)
	Towns and suburbs	1058(38.4)	423(37.4)		1481(38.1)
	Rural areas	471(17.1)	173(15.3)		644(16.6)
Living with children	No	1019(37.1)	499(44.1)	< 0.001	1518(39.1)
	Yes	1730(62.9)	632(55.9)		2362(60.8)
Civil status	Unmarried/divorced/widowed	1488(54.1)	653(57.7)	0.04	2141(55.1)
	Married/ registered partner	1261(45.9)	478(42.3)		1739(44.7)
University level education	No	886(32.2)	520(45.9)	< 0.001	1406(36.2)
	Yes	1852(67.2)	608(53.7)		2460(63.3)
Born in Sweden	No	309(11.2)	133(11.7)	0.634	442(11.4)
	Yes	2446(88.8)	999(88.3)		3445(88.6)
***MS-specific characteristics***					
MS severity^3^	Mild (EDSS = 0-2.5)	1875(68.1)	769(67.9)	0.997	2644(68.0)
	Moderate - Severe (EDSS = 3-9.5)	327(11.9)	135(11.9)		462(11.9)
	Missing	553(20.1)	228(20.1)		781(20.1)
Years since MS diagnosis, median (IQR)		8.2 (4.1–13.5)	7.9 (4.1–12.8)		8.1 (4.1–13.3)
***Working-life characteristics***					
In paid work^4^	Yes	2581(93.9)	1054(93.1)	0.380	3640(93.6)
Current occupation^5^	Employed (yes)	2405(87.3)	936(82.7)	< 0.001	3341(86.0)
	Self-employed (yes)	192(7.0)	161(14.2)	< 0.001	353(9.1)
	Student/work experience placement (yes)	200(7.3)	50(4.4)	0.001	250(6.4)
	On parental leave (yes)	72(2.6)	12(1.1)	0.002	84(2.2)
	On sick leave (yes)	206(7.5)	35(3.1)	< 0.001	241(6.2)
	On disability pension (part-time) (yes)	250(9.1)	47(4.2)	< 0.001	297(7.6)
	Unemployed/Job seeker (yes) or Unemployed/Job seeker (yes)	174(6.3)	70(62)	0.074	244(6.3)
	Other	39(1.4)	17(1.5)	0.838	56(1.4)
	Sick leave/part-time disability pension (yes)	430(15.6)	76(6.7)	< 0.001	506(13.0)
Type of current profession	Managerial responsibilities	295(10.7)	182(16.1)	< 0.001	478(12.3)
	Low to medium physical demands	2177(79.2)	691(61.1)		2872(73.9)
	High physical demands	101(3.7)	173(15.3)		274(7.1)
	Missing/not in paid work	176(6.4)	85(7.5)		263(6.8)
Self-rated importance of paid work^6^	1–2 Least important things	121(4.4)	80(7.1)	0.003	201(5.2)
	3	597(21.7)	241(21.3)		838(21.6)
	4–5 Most important things	2036(73.9)	810(71.6)		2846(67.6)

^1^ Some participants have missing information on type of living area (*n* = 5), living with children (*n* = 7) and civil status (*n* = 7). Thus, not included in the sex stratified nor all study participant columns

^2^ P-values from chi-square tests comparing the proportions for women and men

^3^ From the total sample, low MS severity (EDSS = 0) was observed for 928 participants (23.9%), while more severe MS (EDSS = 6) was observed on 77 participants (2%)

^4^ In paid work assumed from not responding “Not in paid work” in a question asking about one’s professional occupation

^5^ Multiple simultaneous occupations could be reported

^6^ Participants ranked the importance from 1 (one of the least important; 1.4%) to 5 (one of the most important; 27.2%). Numbers do not sum up due to lack of response by some participants

**Abbreviations**: EDSS: Expanded Disability Status Scale; IQR: Interquartile range; MS: Multiple sclerosis

Paid work was reported by 93.6%. Most were employed (86.0%), with women (87.3%) more often being employees than men (82.7%). In contrast, a higher proportion of men (14.2%) than women (7.0%) reported self-employment. Sick leave or part-time disability pension was reported by 13.0%. Most PwMS had professions characterised with low-medium physical demands (73.9%). Paid work was frequently rated as one of the most important things in the participants’ lives (27.2%), and 67.6% reported work as important.

### Impact of the pandemic on one’s working life

The reported impacts of the pandemic on one’s occupation are contained in Table 2. Notably, 2731 (70.3%) responded “No impact”. Overall, 26.2% of the participants reported an impact on their occupation by the pandemic. No differences were observed by sex or MS severity, but differences were observed by educational level and by type of current profession among those reporting a particular impact.

**Table 2:** Impact of COVID-19 on one’s occupation for all study participants and stratified^**1**^ by sex, MS severity, education level, and type of current profession

	By sex		By MS severity (EDSS scores)			By level of education		By type of current profession			All study participants^2^
	Women	Men	0-2.5	3-9.5	missing	Primary or high school	University	managerial responsibilities	Low-medium physical demands	High physical demands	
	*n* (%)	*n* (%)	*n* (%)	*n* (%)	*n* (%)	*n* (%)	*n* (%)	*n* (%)	*n* (%)	*n* (%)	*n* (%)
**n participants (% of all)**	2755(70.7)	1132(29.1)	2644(68.0)	462(11.9)	781(20.1)	1406(36.2)	2460(63.3)	478(12.3)	2872(73.9)	274(7.1)	3887 (100)
**No**	1954(71.1)	772(68.3)	1867(70.6)	325(70.4)	539(69.0)	950(67.6)	1768(71.9) *	351(73.4)	2070(72.1)	173(63.1) *	2731(70.3)
**Yes** ^**3**^	737(26.8)	279(24.7)	688(26.0)	123(26.6)	207(26.5)	394(28.0)	617(25.1) *	104(21.8)	719 [25]	62(22.6) *	1018(26.2)

^1^ P-value for difference in proportion responding calculated with chi-square tests, * represents a p-value < 0.05

^2^ Individuals with missing information are excluded from the stratified columns but included in the total study population: *n* = 781 missing EDSS scores, *n* = 21 missing information on their educational level, and *n* = 263 missing information on their type of profession (of which 247 were not in paid work)

^3^ Number of participants reporting at least one impact to their occupation in the closed-answer options below. The closed-answer options are not presented given small cell counts in some strata

**Abbreviations**: EDSS: Expanded Disability Status Scale; MS: Multiple sclerosis

There were differences observed concerning the type of impact that the pandemic has entailed. Regarding interruptions to paid work, 7.0% of the participants were or had been furloughed. Higher proportions of men than women (9.1% vs. 6.2%) and participants without university education (9.0% vs. 5.9%) reported furlough. Among all participants, 2.8% reported unemployment. Although no differences in unemployment were detected by sex, higher proportions were reported among participants without university education (4.2% vs. 1.9%).

Being in work with more to do was more frequently reported than having less to do (8.8% vs. 3.0%). The impact of the pandemic on one’s workload differed by sex, educational level and type of profession (see Table 2).

### Self-reported impacts of the pandemic on one’s working life

The qualitative analysis identified four categories in how the pandemic had affected the participants’ working lives (Table 3) and renamed as the following:

**Table 3:** Overview of categories and sub-categories of content of the impact of the pandemic on people with MS’ working life

Category of content	Subcategories
**Direct impact on one’s occupation**	• Relating to work capacity
	• Consequences of working from home
	• Adaptations within the same occupation
	• Stops and interruptions to paid work
	• Improved workplace relationships
**Disclosing or concealing MS in the workplace**	• The pandemic as a driver to disclose one’s MS
	• Negative consequences of concealing MS diagnosis during the pandemic
**Worry and uncertainty**	• Worry about one’s own consequences
	• Others’ worry for the person with MS
	• Sense of uncertainty and frustration
**Broader impact to life situation**	• Personal wellbeing
	• Social and family relationships
	• Economic

**Abbreviations**: MS: Multiple sclerosis

***Direct impact on one’s occupation.*** The first category regarded the direct impacts the pandemic had on one’s daily occupation. These impacts largely corresponded to the findings from the results of the close-ended questions, but also give a deeper understanding of how the pandemic had affected the participants’ daily occupations and consequently their wider working life. The direct impacts were further organised into five subcategories.

*Relating to work capacity.* The first subcategory comprises statements of one’s work capacity during the pandemic. Several participants reported reduced work capacity due to acute COVID-19 infections or post-COVID symptoms. One woman wrote:

*“I am so much worse now after my covid infection than before, but since I work in an industry where there have been hardly any jobs* […] *But it’s much more difficult to do the jobs I do now and I’m bed-bound several days afterwards, so if the world had been as usual, I would have to deal with this …”* – Woman aged 40–49, missing EDSS.

Reduced work capacity and increased sick leave during the pandemic, among both respondents and their colleagues, were described as underlying the greater workload often experienced during the pandemic.

However, several participants stated that they better maintained their work capacity during the pandemic. Such statements elaborated that they had less sick leave, often owing to fewer infections with seasonal colds because of their self-isolation and the heightened awareness of hygiene in society.

*Consequences of working from home.* A reoccurring reported impact of the pandemic to one’s current occupation was the shift to home or distance work/studies to mitigate the risk of infection. For some, this new possibility to work remotely (full or part-time) was seen as positive. It was also positive for participants already working from home, as this now had become a shared work experience. Other positive features included increased productivity in work and private life, calmer and more controllable work environment and working schedule, and saving time and energy from commuting. Accordingly, a key finding was that working from home was a strategy for the participants with MS to manage energy levels and life balance. As one participant reported:

*“To work from home has meant that I could control pressure, sound level, moments of interruptions etc. in another way than if I sat at my workplace. I have been able to plan my working day/week according to my current state*, […] *the lack of commuting has given me more balance in life and less stress.”* – Woman aged 30–39, missing EDSS.

Related to these positive aspects, several wrote that they hoped to be able to continue working from home. Nonetheless, feelings of isolation and low mood were often reported in relation to working from home during the pandemic. New work challenges were also highlighted, including having small children at home, new technology, alongside some aspects of work, such as communication and problem solving, becoming more difficult.

*Changes within the same occupation.* This subcategory provided details on the adaptions to and disruptions within one’s current occupation during the pandemic. Adaptions in this subcategory were those not working from home. The statements of participants working onsite often contained reactions to adaptations to mitigate risk of infections, for example, working outdoors, protective clothing, or reassignment within the organisation. Some workplace changes due to COVID-19 were reported as incompatible with their MS and resulted in increased sick leave, for example, one participant shared:

*“Had to work at another workplace when my usual one was forced to close due to covid. The temporary workplace is not at all adapted to my problems; therefore, I have unfortunately been on sick leave to a larger extent than if I had been at my regular place of work.”* – Woman aged 40–49, EDSS = 3.

Accordingly, this subcategory also reflects situations where the participants could not adapt to the changing work situation during the pandemic due to their MS.

Participants also reported changes in the quantity or type of work tasks. New or changed work conditions or assignments were described as downstream impacts of the pandemic on wider industry standards and consumer demands.

Disruptions included cancelled business travels but also difficulties in completing studies with regards to placements and work experience.

*Stops and interruptions to paid work.* This subcategory included participants reporting that they were furloughed, unemployed, job seeking, lost work or did not get their contract renewed, or quit their job during the pandemic. Several participants also indicated that they found it harder to get a new job during the pandemic and that there were less options suitable for their MS.

Many of the interruptions and stops to their daily occupation were related to self-isolation. Several responses described that the income support for self-isolation came from the various social insurances and allowances related to health including sickness absence benefits, carrier allowance, and the risk group allowance. These statements focused on the interruption or stops of their working life and were not related to a change in their work capacity. Several participants stated they were eligible for income support owing to their MS treatment. Some PwMS also stated that they considered themselves at risk for COVID-19 and chose to stop working to self-isolate. These participants could not work from home or maintain physical distance onsite and were not eligible for the risk group allowance to self-isolate. Participants disclosed that this was financed privately through own savings or family support. One participant who used parental leave for this purpose shared:

*“My employer could not offer work from home and I was denied compensation from the Social Insurance Agency because MS is not included in the risk groups, so the father of my children was forced to give his parental leave days to me.”* – Woman aged 40–49, EDSS = 1.

*Improved workplace relationships.* The last subcategory concerned experiencing improved understanding and support in one’s workplace relationships. This more positive impact of the pandemic was exemplified in this statement:

*“Increased understanding from my boss during the periods when I have been worried because of a treatment swap or when I avoided going to the office because of the pandemic and risk of infection.”* - Woman aged 30–39, EDSS = 1.

Improved relationships with co-workers were also reported as were increased understanding of their change in working location or extent of work during the pandemic.

***Disclosing or concealing MS in the workplace.*** The second category included statements about the disclosure or concealment of MS diagnosis in the workplace during the pandemic, and was organised into two subcategories.

*The pandemic as a driver to disclose one’s MS.* Responses about new disclosures of one’s MS diagnosis in the workplace were clustered around three points in the pandemic: The pandemic breaking out, introduction of vaccines, and return to onsite work. The disclosures were often more forced early in the pandemic, as one woman shared:

*“In connection with the pandemic I felt forced to tell that I belonged to a risk group and needed my employer’s support to work from home. Before this, I had the approach to never disclose unless the disease became active or worse and affect my work capacity.”* - Woman aged 30–39, EDSS = 1.

Disclosing MS to one’s employer was often done in response to direct questions about staff belonging to a risk group, to address their worry about being infected at work, to explain sick leave or vaccination choices, to motivate continuation of remote work, or to enable other work adjustments. Positive outcomes from disclosing to one’s employer were often reported. Although a few also experienced negative consequences, the feeling of being a “problematic employee” was often expressed in these cases.

In contrast, disclosing one’s MS to co-workers was more often reported to have come up naturally in general discussions about COVID-19, changes in work tasks, but also to explain one’s vaccination choices as the below statement highlights:

*“Even though I am the youngest at work I was invited to be vaccinated first (this was to match the timing for my next treatment as good as possible). It sounded strange to be vaccinated so early at my age, so felt that it needed explaining.” -* Woman aged 20–29, EDSS = 0.

Later in the pandemic, several participants disclosed their diagnosis to their employer to enable the continuation of the work adaptations initiated for the pandemic that were beneficial for their MS. While these statements often related to working from home, more general discussions on adaptations and support were noted to be facilitated through these disclosures.

*Negative consequences of concealing MS diagnosis during the pandemic.* Some decided to conceal their MS diagnosis, which was reported to have had some negative consequences in relation to the pandemic. For example, participants were aware they were working in risky situations which could have been avoided, found it hard to motivate continuation of adaptations to the pandemic, or had to provide vague comments regarding their vaccination status. One woman working within healthcare wrote:

*“I have met and investigated many covid-positive patients despite that I consider myself to be in a risk group as well as unvaccinated. This could have been avoided if I told my manager about my disease.”* - Woman aged 30–39, EDSS = 0.

In addition, participants reported misunderstandings among their co-workers regarding their actions or non-participation in activities. No positive consequences from concealing MS at the workplace were reported regarding the pandemic.

***Worry and uncertainty.*** The third identified category was worry and uncertainty triggered by the pandemic. This had implications for the participants’ work situation, but also spanned wider aspects of life and was organised into three subcategories.

*Worry about one’s own consequences.* The first subcategory related to the participants’ worry for themselves during the pandemic. This was often described as related to fear of infection and was a constant mental load which was exhausting and brought their life to a standstill. Anxiety of belonging to a risk group was also mentioned to arise in situations when they could not manage the risks or control their distance to other people at the workplace, while commuting, or out in public more generally.

Furthermore, there were several statements expressing worry in relation how the pandemic was perceived to have worsened their future prospects. Society opening and the risks COVID-19 would pose then were reoccurring aspects. These statements often lifted worries about their MS and treatments as well as wider life changes. One man wrote:

“*The biggest change that the worsening of my MS is having on work and life is the ever-growing fear of the future. I am fearful of not working, of losing my job, of not being able to provide for my family, for not being able to have the future with my children that I imagined.* […] *Recently during covid times, it has gotten a lot worse. Couple this with the news that I likely won’t be walking in 10 years’ time and the rapid decline in my mobility. I’m fearful of the future and it affects me every day and I have no way to change that. I am bound, trapped but must keep going. It creates a mental exhaustion that is honestly worse than the physical one…”* – Man aged 40–49, EDSS = 1.

*Others’ worry for the person with MS*. Others’ worry for them during the pandemic was another subcategory. Participants who had disclosed their MS in the workplace sometimes felt unwelcome at work by their worried employer and were forced to isolate. Others received comments of concern from co-workers. As one participant reported:

*“When COVID came, I got a few comments such as “I don’t get that you dare to go to work, you can be very sick”* […] *Surely out of concern, but I do not need other’s opinions on whether or not I can work.”* - Woman aged 40–49, EDSS = 0.

*Sense of uncertainty and frustration.* A sense of uncertainty and frustration with the pandemic was also expressed. This had implications for the participants’ work participation and mental load. Uncertainty often related to the lack of or changing knowledge about COVID-19 and the risk of severe sequelae. Frustration in relation to mixed messages as to whether PwMS were considered a risk group were apparent in several responses, one woman elaborated on her feelings about this:

*“Risk group or not? So many different messages, different from other countries. If I can NOT keep my distance at work, what rights do I have? At times masks prohibited, not prioritised for vaccine. MS with other, e.g. overweight - means risk group. But not according to rules.* […] *A doctor says that you are a risk group, but that FK* [Swedish Social Insurance Agency] *will not approve sick leave. Considered changing profession when one was not protected. Many teachers sitting in the same boat. Short-term solutions with contagion allowance have meant that many have drawn from their savings and felt very bad during corona…”* – Woman aged 40–49, missing EDSS.

***Broader impact to life situation.*** The last category reflects both positive and negative aspects of a broader change in life situation during the pandemic, relating to personal wellbeing, social and family relationships as well as economic situation. These aspects of life were described as connected to, and interplaying with their working life, and vice versa in respect of their experiences of the pandemic. One woman who reported that she was re-entering work owing to the household’s changed economic situation wrote:

*“I have been a housewife for some years, which has been very good for my MS. I have been able to do everything at my own pace and life has been very positive. Unfortunately, my husband became unemployed due to the corona-pandemic and I therefore have had to rethink and try to find a way to get back into working life …”* – Woman aged 40–49, missing EDSS.

### The pandemic’s impact on self-employed participants

There were 353 participants who were self-employed, of which 62.0% had university education and their median age was 43 years (IQR: 38–47) (data not shown).

The majority of the self-employed participants (68.3%) reported being able to work mostly as usual during the pandemic (Table 4). Overall, 33.7% reported that their business has gone better than before. Digitalisation of business activities and taking other types of assignments were more frequently reported than having a dormant or bankrupt business. Nonetheless, 43.9% reported “no” to the statement “My business has gone better than before”.

**Table 4:** Self-employed participants with MS responding yes/partly to the following statements about how COVID-19 affected their business activities

How has the ongoing COVID-19 pandemic affected you in respect of being self-employed?	Proportion of self-employed participants reporting yes/partly
	%
My business has gone better than before	33.7
I have mostly been able to work as usual	68.3
I have digitalised my business activities	33.4
I have undertaken other types of assignments	20.4
My business has gone down, but I have managed to keep it going	35.4
The business is dormant / I have dissolved my business / My business has gone into bankruptcy	12.5

**Abbreviations**: MS: Multiple sclerosis

Self-employed participants with MS (*n* = 353)

Regarding the questions posed to all participants about the impact of the pandemic on one’s occupation, lower proportions of the self-employed reported an impact on their occupation (15.6%) (data not shown).

## Discussion

Impacts of the COVID-19 pandemic on PwMS’ working life were investigated in this mixed-methods study from Sweden. The participants often described that they regarded themselves at risk during the pandemic and that they had to navigate changing systems and recommendations. Overall, most participants reported no impact, with 26.2% reporting a particular impact of the pandemic to their occupation. This reasonably low proportion could be due to the pre-existing challenges that they may face in their working life with MS. Differences were observed among those reporting an impact by sex, type of profession, and level of education, but not by MS severity. Four categories of impact of the pandemic on working life were identified in the qualitative analyses: *Direct impact on one’s occupation*; *Disclosing or concealing MS in the workplace*; *Worry and uncertainty*; and *Broader impact to life situation.* The findings from the open-ended questions supplemented the results from the closed-ended questions, describing the accumulation of challenges and the additional layer of uncertainty during the pandemic in relation to having MS.

Work was frequently reported to be an important aspect of the participants’ lives. Yet, the pandemic and accompanying mitigation measures had wide impacts on the participants’ working lives. The most evident category of direct impact of the pandemic identified was a stop or interruption to paid work. Under normal circumstances, there can also be various transitions within one’s work trajectory, but the pandemic had profound impact on work participation [28], including for PwMS. Transitions to unemployment may have repercussions for one’s livelihood, finances, and quality of life [29, 30]. Among the participants, 5.8% were unemployed or job seekers and 2.8% reported that they were unemployed as an effect of the pandemic in a later question. The impact of the pandemic has been unequally distributed, including in Sweden [31]. Higher proportions of participants with professions with high physical demands or less education reported unemployment. This is likely related to the different jobs that qualifications enable and the distribution of these jobs across the sectors affected by the pandemic. While there were no differences in the proportions of men or women reporting unemployment, more individuals without university education and men reported being furloughed, which corresponds with wider trends in Sweden of a higher proportion of furloughed men than women (9.7% vs. 6.5% in May 2020) [31]. These observations are likely due to the vast gender differences in the Swedish labour market, with higher proportions of women in professions orientated towards people [32, 33]. Working with people is an important job characteristic regarding the risk of transmission but also often continuing to operate during the pandemic (e.g. nurses or teachers). In contrast, professions orientated towards creation of objects (e.g. manufacturing or construction) were often paused. It is widely suggested that the aftermath of the COVID-19 pandemic may result in different trends than previous recessions, affecting more women and younger individuals [31, 34]. Nonetheless, we were unable to discern clear trends of this, nor could a previous study specifically investigating gender impacts in Sweden [31]. The authors, partially attributed this to primary schools and kindergartens never fully closing, as in other countries, and that professions in high demand during the pandemic, like nursing, have a largely female workforce [31]. Lastly, finding not only a job, but one suitable for their MS was an additional layer of complication experienced by the job-seeking participants. Therefore, suggesting more limited work options suitable for MS during the pandemic.

The majority (68%) of the self-employed participants reported being able to work mostly as usual during the pandemic. Many adapted their business to the changing landscape by rapid digitalisation or taking other assignments. That said, under half reported “no” to the statement that their business was going better than before, with more individuals reporting their business was dormant than dissolved or bankrupt. This could have indicated a temporary impact of the pandemic or that decisions to discontinue and the resulting administrative processes took time [35]. Today, we can assume that these statistics may reflect a period of uncertainty, during which businesses faced difficulties but were able to stay afloat due to economic support from the government. Accordingly, our findings could not have fully captured the long-term impact of the pandemic, initially resulting in an underestimation, given that bankruptcies increased significantly after the summer of 2022 [36]. Alternatively, self-employed PwMS may already have had plans for interruptions to their business activities from their MS and may have been more resilient to disruption.

A unique impact of the pandemic were the work interruptions to self-isolate to avoid infection. One study modelling the spread of COVID-19 concluded that large portions of the population in Sweden voluntarily self-isolated [37] with another study comparing residents in Sweden and Norway observing that most Norwegians (88%) and Swedes (74%) stayed at home during their spare time [15]. Similarly, the PwMS in our study reported that they were proactive in changing their behaviour to reduce their infection risk and reduce transmission, highlighting the importance of both public health recommendations and individual control measures in the pandemic. Nonetheless, confusion and uncertainty as to whether PwMS constituted a risk group as well as dismay with the official definition excluding MS were reported. The findings indicated that this confusion resulted in worry in being in direct contact with people in general, including at the workplace, as all people were possible carriers of COVID-19. Worry was also associated with increased social isolation of the participants. Accordingly, worry was underlying many of the participants’ choices to pause their working life to self-isolate and was expressed by the participants to span many aspects of life. Concerns about susceptibility and risk of severe COVID outcomes was also reflected in their descriptions of how employers and co-workers responded to their disclosure of MS. Several participants reported anxiety and worry in relation to the pandemic in general as well as working onsite during the pandemic, owing to their job being unable to be performed remotely or from concealing their diagnosis from their employer. Several participants unable to negotiate remote work received social insurances to replace lost earnings when self-isolating, often owing to their immunosuppressing MS treatment. The responses also included some participants, ineligible for financial assistance, who chose to end their employment to self-isolate. Therefore, taking this decision at their own or at their family’s cost or using an alternative social insurance (e.g., parental leave) to achieve this purpose. This is despite most participants rating work as one of the most important things in their lives. The Swedish approach of voluntariness and self-regulation may have provided flexibility and sustainability over longer periods of time [37] but also less solidarity with individuals perceiving themselves as at risk but not squarely fitting within the definitions to access support [35]. While many interruptions to one’s occupation during the pandemic were imposed upon the participants, for some PwMS, stopping or pausing their working life was their solution to the new challenges and risks of working during the pandemic.

The pandemic has been a catalyst for rapid change in how we work [28]. Changes to one’s current working conditions were also reported in our study. Similarly, a cross-sectional survey of PwMS in the USA found that 37% of participants reported changes to paid work because of COVID-19 [38]. The most common change reported was the same as identified by our participants, that they now work from home [38]. Other changes reported included switching shifts, cancelling travel, reductions in salary, and leaves of absence [38]. In contrast, change of employment wasn’t a mayor reaction to the impact of the pandemic among our participants, with only 4.4% reporting having changed jobs and 1.8% indicating some degree of change (data not shown). Regardless, experiences of the vast changes can provide new knowledge and strategies to utilise remaining work capacity among workers with chronic diseases going forward.

The ability to work from home was largely reported to be positive and led to an increase in perceived work productivity. Some participants related this to reduced commuting which saved time and energy, more flexibility in working hours, or increased ability to control their work environment. One could assume that working from home might also alleviate concerns about infection risks, potentially reducing constant uncertainty and indirectly enhancing work productivity. Regardless, working from home is often associated with greater flexibility in deciding, when, where and how to work [39]. With a disease characterised with uncertainty and fatigue [40–42], flexibility and control are crucial. Of note, many expressed their wish to continue working from home, at least partially. However, not all jobs lend to working remotely, with women more likely than men to have compatible jobs [14, 31, 43]. Large differences have also been observed in the proportions of workers in Sweden working from home between industries, and geographical areas (higher proportions in urban areas [43]. Further aspects such as the home environment can also affect the suitability of remote work [14], which was highlighted by several participants expressing the new work challenges with small children at home. Accordingly, while not applicable for all, this increase in remote work is a promising tool for many PwMS to maintain their work participation. Potential risks of increased remote work have been increasingly discussed, including risks for one’s career as well as wider challenges of blurred work-life boundaries and ergonomics [14, 39, 44]. Working from home was facilitated by a rapid digitalisation of work [14, 39]. This can be seen with a third of our self-employed participants digitalising their business activities. The increase in technical solutions and normalisation of remote work may facilitate work, especially among people with disabilities [14]. Accordingly, our study adds to the discourse on the benefits of adequately organised remote work as a tool to manage energy and to facilitate work participation among PwMS. The trend of increased hybrid working and normalcy of working from home is a likely benefit in the years ahead for PwMS or managing work with another chronic disease.

The transition back to working onsite can be complex. Indeed, this was often a driver for the participants to disclose their diagnosis to motivate the continuation of adaptations initiated because of the pandemic. Disclosure to the employer was one way of navigating the changing situation and addressing their worry. Worry for the future and society opening were also commonly reported. Return-to-work and easing lockdowns were observed to be associated with reduced mental health among PwMS in Italy [45]. Concealment of one’s MS is common and facilitated by the often-invisible symptoms, but individuals who conceal their diagnosis may be deprived of accommodations to facilitate work participation [46]. Concealment may also have negative consequences on psychosocial outcomes [47] and cognition by constantly allocating brain resources to conceal one’s MS [48]. Mistaken and biased opinions about MS and a lack of MS knowledge were lifted in the responses of our participants regarding the negative consequences of disclosure, however, others reported improved understanding and support in the workplace. Further studies following these new disclosures during the pandemic are needed as well as continuous support for PwMS with their ongoing challenge of disclosing symptoms as they emerge [48].

COVID-19 started as a health crisis, but with time, came economic challenges from self-regulation and restrictions and it has morphed into a societal crisis [14, 28, 35]. Workplaces, including workers, have navigated the pandemic largely by improvisation before these new work procedures became routine [14, 28]. The uncertainty of the pandemic, changes in employment, and working conditions may have longer-term impacts as the pandemic has already accentuated pre-existing labour market inequalities and heightened the risk of exclusion among many [28]. Therefore, the recent trend of progress in improving work outcomes among PwMS may be reversed [8, 49].

### Methodological considerations

This study has several strengths, including the large population-based sample of working-aged PwMS and the subsequent linkage of their survey responses with high-quality register data [23, 24]. The online survey had a response rate of 52%, potentially reflecting the convenient mode of administration allowing for completion of the survey in one’s own time and chosen environment [50]. Nonetheless, men and PwMS born outside of Sweden responded to a lower extent which could affect the nature of the impacts reported.

A high proportion of our participants had an EDSS score of 0 (24%). These participants likely had some impaired functioning, despite scoring below the traditional clinical threshold [51, 52]. It is important to investigate outcomes in early MS as well as how to utilise and maintain remaining work capacity. Nonetheless, this relatively mild impairment of neurological function should be considered when interpreting our findings.

Limitations to consider include the cross-sectional design, reducing our ability to conclude on the causality of the pandemic. Additionally, we do not know how non-responders to the survey were impacted by the pandemic.

Further, the survey was administered during the pandemic, thereby reducing possible recall of the impacts on one’s occupation but possibly underestimating the long-term impacts. However, we have yet to observe the long-term consequences or longevity of some of the impacts already experienced. The post-pandemic era will illuminate which interruptions are better classified as a pause or labour market exit.

## Conclusions

Among the quarter of participants with MS reporting an impact, the consequences of the pandemic on their occupation differed. Uncertainty and worry were often underlying the participants’ responses. Working or studying remotely was the most reported change and it was often described as conducive for their energy levels and productivity. Many expressed a wish to continue working from home, even disclosing their MS to their employer to motivate it. The participants improvised during the pandemic to find solutions to continue or pause their working lives in the context of uncertainty and change. It is important to ensure the positive learning experiences of the pandemic of factors promoting work are implemented and explored going forward alongside supporting individuals who paused their working lives during the pandemic back into work. The lessons learnt from changed working conditions need further exploration to support and facilitate the working goals of people with functional limitations going forward. The present study adds the perspective of PwMS to the discussion. The experiences and lessons of the pandemic should not be wasted.

## Data Availability

The data in this project is not publicly available in accordance with the General Data Protection Regulation, the Swedish Data Protection Act, the Swedish Ethical Review Act, and the Swedish Public Access to Information and Secrecy Act. Readers may contact Associate Professor Emilie Friberg (emilie.friberg@ki.se) regarding the data.

## Abbreviations

EDSS: Expanded Disability Status Scale
IQR: Interquartile range
LISA: Longitudinal Integrated Database for Health Insurance and Labour Market Studies
MS: Multiple sclerosis
PwMS: People with multiple sclerosis
SMSreg: Swedish Multiple Sclerosis Registry
